# Seated Virtual Reality-Guided Exercise Improved Gait in a Patient With Trunk Dysfunction Due to Hip Fracture: A Single-Case Design Study

**DOI:** 10.7759/cureus.62433

**Published:** 2024-06-15

**Authors:** Kengo Kawanishi, Masami Nakamoto, Toshinori Mityashita, Seiji Ogita, Shintatou Kudo

**Affiliations:** 1 Inclusive Medical Sciences Research Institute, Morinomiya University of Medical Sciences, Osaka, JPN; 2 Emergency Center, Kano General Hospital, Osaka, JPN

**Keywords:** reach exercise, gait, virtual reality, hip fracture, trunk dysfunction

## Abstract

Physical therapy for mobilization after a hip fracture is effective in improving muscle strength and balance training of the lower extremities; however, effective interventions targeting the trunk muscles remain to be established. The efficacy of virtual reality (VR)-based exercise therapy has been recently reported. This case report demonstrates the effectiveness of VR-based intervention in improving the gait of a female patient in her 90s with a femoral neck fracture who had difficulty in independent gait postoperatively due to trunk dysfunction.

The patient had undergone bipolar hip hemiarthroplasty for a right femoral neck fracture sustained via a fall at home. Standard physical therapy, including range of motion exercises, resistance training, and gait training, was commenced gradually the day after surgery. An improvement in lower extremity pain was reported at the two-month follow-up visit but without any improvement in the gait ability. Trunk flexion was observed during gait, and the patient had difficulty in independent gait and walking without upper limb support. Withdrawal and reversal designs (BAB) were employed, and each period lasted one week. Standard physiotherapy supplemented with trunk reach training in a seated position using VR equipment was commenced subsequently. An improvement in the maximum anterior pelvic tilt angle and sitting and standing postures, increased hip extension range of motion and step length during gait, and decreased gait speed were observed during the intervention phase. These results highlight the importance of physiotherapy interventions targeting the trunk muscle and the effectiveness of VR-guided trunk training in patients with femoral neck fractures.

## Introduction

The incidence of hip fractures is on the rise in Asia [1], and 33% of patients with hip fractures are unable to attain their pre-injury mobility level postoperatively [2]. Furthermore, the incidence of secondary fractures, such as contralateral hip fractures, has been reported postoperatively in 28.6% of patients with hip fractures [3]. Hip fractures have been frequently reported among older adults. Lower extremity muscle weakness [4,5] and trunk muscle weakness [6] are often observed before injury. Thus, besides lower extremity muscle problems, trunk muscle atrophy and other trunk dysfunctions may inhibit the return to pre-injury gait, leading to a vicious cycle of repeated falls and secondary fractures after sustaining the initial hip fracture.

Previous studies have reported the effectiveness of physical therapy for gait training after a hip fracture in improving muscle strength and balance training of the lower extremities [4,5]. However, effective interventions targeting the trunk muscles remain to be established. Trunk muscle training in supine and side-lying positions improves the gait ability of community-dwelling older adults [6,7]. However, it is difficult for older adults to assume supine and side-lying positions after hip fracture surgery, thereby hindering their application in clinical settings.

The effectiveness of virtual reality (VR)-based exercise therapy has been reported in recent studies [8]. The beneficial effects of mediVR KAGURA® (mediVR, Inc., Osaka, Japan), which involves reaching exercises targeting the trunk muscles in a seated position, on gait function have been reported in patients with cerebrovascular disease and postoperative patients with hallux valgus who have difficulty maintaining a stable gait [9,10]. We discuss a case of a patient who showed improvement in independent gait following a physical therapy intervention using mediVR KAGURA®.

## Case presentation

The patient was a female in her 90s who had undergone bipolar hip hemiarthroplasty for the treatment of a right femoral neck fracture sustained via a fall at home. She had sustained multiple fractures via falls in the preceding few years, including fractures of the seventh, 11th, and 12th thoracic vertebrae; fracture of the fifth lumbar vertebra; fracture of bilateral distal radius; and fractures of the distal phalanges of the right big toe. The patient had undergone total knee arthroplasty for the treatment of bilateral osteoarthritis of the knees. She had resided alone until hospitalization for the treatment of the right femoral neck fracture. She used a walker indoors for ambulation and a wheelchair outdoors with assistance.

**Figure 1:**
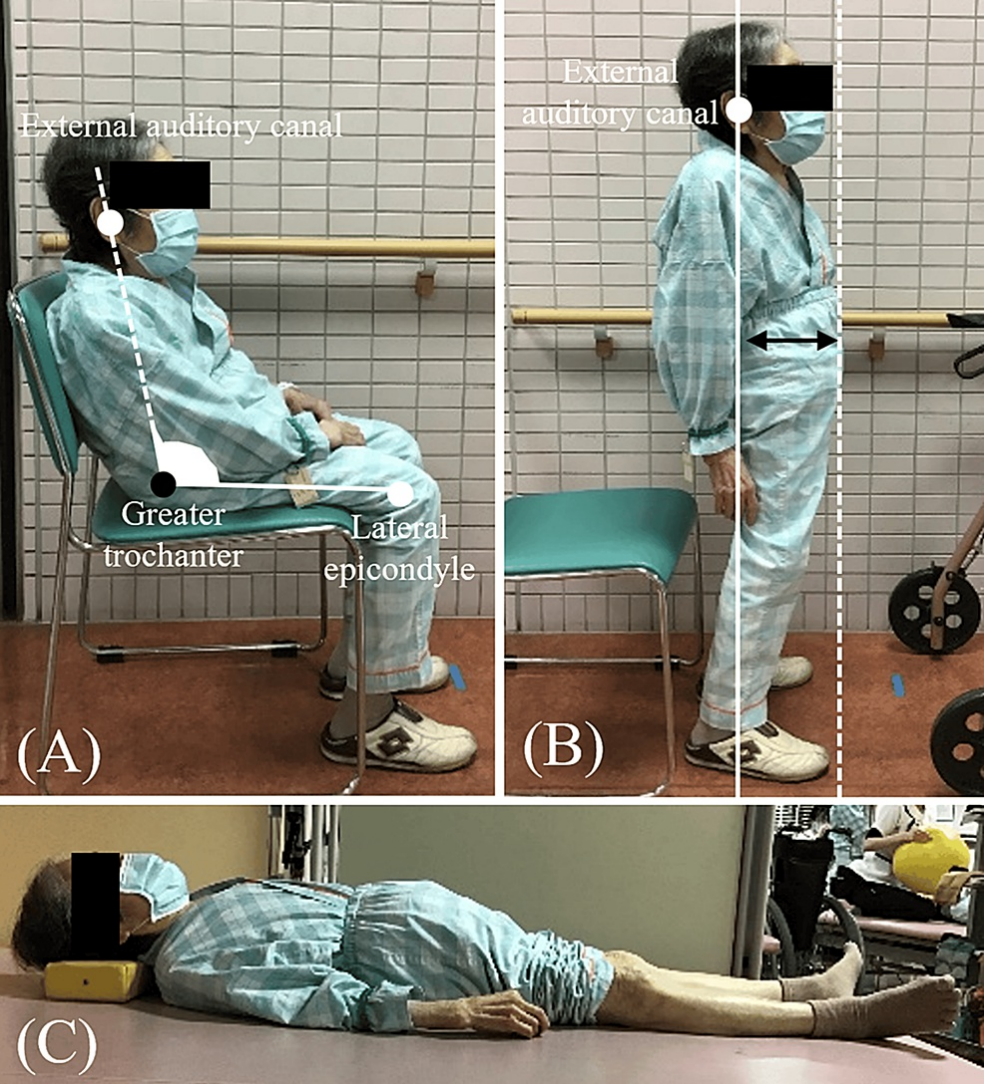
Posture alignment before the intervention (A) Sitting posture and pelvic tilt angle. (B) Standing posture and pelvic sway distance. (C) Supine position The pelvic tilt angle: the angle between the line connecting the external auditory canal to the greater trochanter and the line connecting the lateral epicondyle to the greater trochanter. The pelvic sway distance: the distance between the vertical line of the external auditory canal and the maximum point of pelvic sway

Standard physical therapy, including range of motion exercises, resistance training, and gait training, was commenced the day after surgery. The maximum 10-m walk time (10 MWT) improved to 18.4 s with the walker one month postoperatively. An improvement in lower extremity pain was observed at the two-month follow-up visit; however, no improvement in the 10 MWT was observed. The trunk flexion was observed during gait, and the patient had difficulty in maintaining independent gait and gait without upper limb support. Kyphosis alignment was noted in the antigravity position (seated position); however, she was able to maintain an upright posture in the degravity position (supine position). Minimal deformity of the spinal structure and a decrease in trunk muscle function were observed (Figure 1).

Investigations

**Figure 2:**
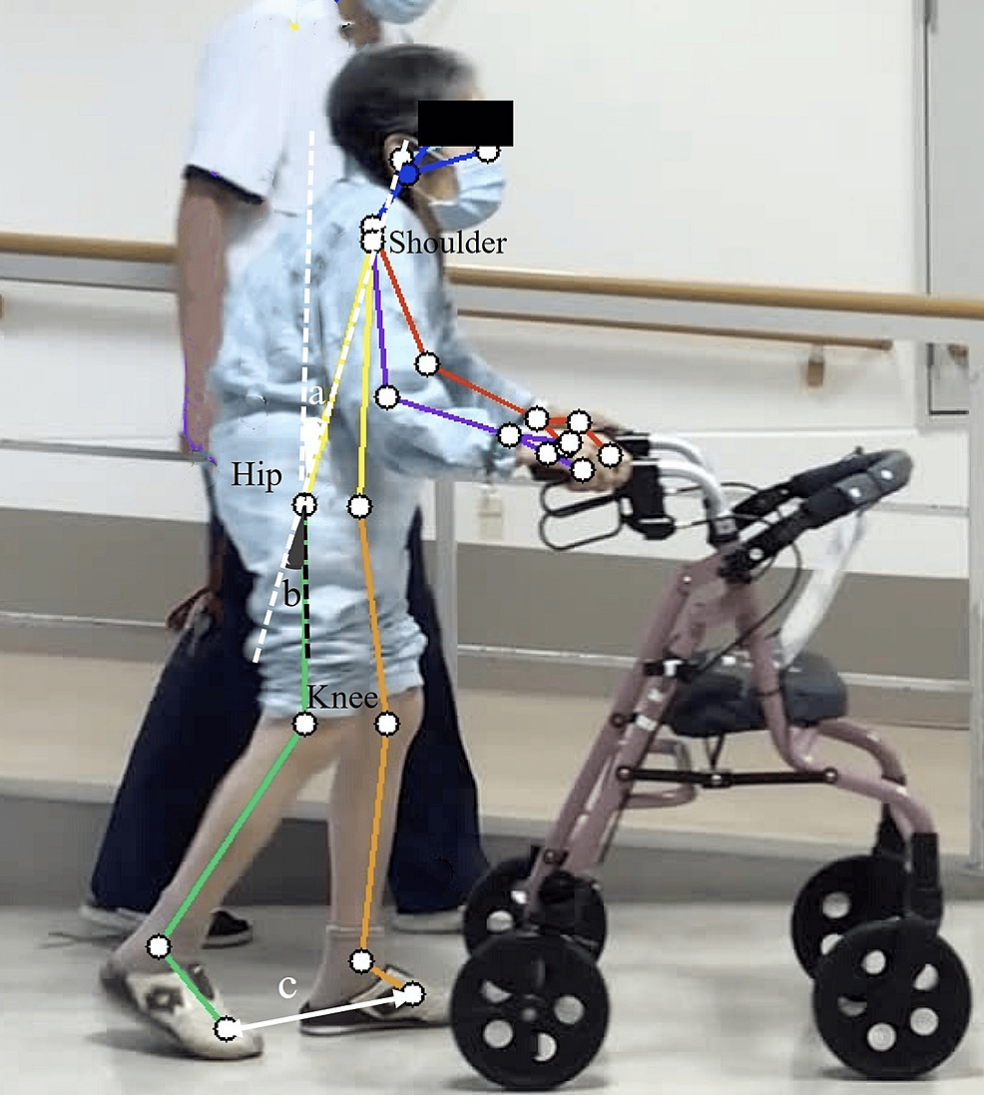
Assessment of alignment during gait using Pose-cap a: Trunk anterior tilt angle. b: Hip extension angle. c: Step length The trunk-tilt angle: the angle between the vertical line of the hip joint and the line connecting the shoulder and hip joints. The hip extension angle: the angle between the lines connecting the shoulder joint to the hip joint and the hip joint to the knee joint

Withdrawal and reversal designs (BAB) were followed, and the duration of each period was one week. The improvement was assessed visually during the non-intervention period (period A) based on the standard of improvement. The 10 MWT and number of steps at maximal effort were measured as gait assessments before and after each period. The hip extension angle, trunk tilt angle in the terminal stance phase, and step length were calculated as the average of three gait cycles using Pose-cap, a markerless skeletal recognition software program (Four Assist Co., Ltd., Tokyo, Japan) (Figure 2).

Postural assessments were performed using ImageJ software Version 1.48 (National Institutes of Health, Bethesda, MD). The angle of pelvic tilt in the seated position (seated pelvic tilt; Figure 1A) and the distance of pelvic shift in the standing position (standing pelvic shift; Figure 1B) were measured. The cross-sectional area of the multifidus and psoas major muscles and the thickness of the external oblique abdominis, internal oblique abdominis, and transversus abdominis muscles were measured via sonography (Aplio α Verifia, Canon Co. Ltd., Tokyo, Japan) to evaluate trunk function. The angle of maximum pelvic anterior tilt in the seated position was measured using a smartphone application (Simple Angle Meter; Neko-system, Osaka, Japan). The Five Times Sit to Stand test, functional reach test, and dynamic balance assessment using the Berg Balance Scale were conducted to assess the performance. In addition, the range of motion of the joint (hip flexion and extension, knee extension, and ankle dorsiflexion), muscle strength (hip extension, hip abduction, and knee extension), and maximum lower-extremity weight shift in the standing position were assessed to evaluate lower-extremity function.

Treatment

**Figure 3:**
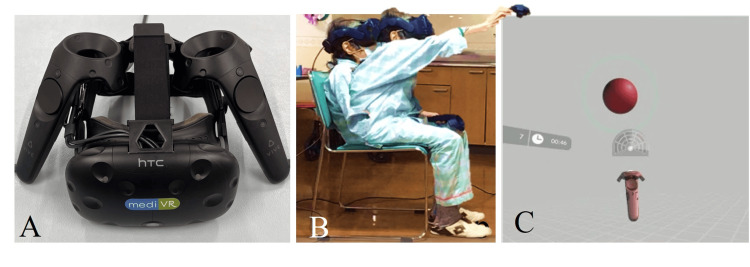
Virtual reality equipment and interventions A: Virtual reality equipment. B: Intervention performed in virtual reality. C: The patient's view of the virtual reality space Image credits: Kengo Kawanishi

Standard physical therapy interventions were performed twice daily for 40 minutes to improve trunk flexion during gait and reduce walking time. Trunk training, comprising anterior-posterior pelvic tilt exercises and upper limb raising, was performed during Phase A. Seated reaching training was performed using VR equipment (mediVR KAGURA; mediVR, Osaka, Japan) in Phase B for 20 minutes once a day (Figure 3). Objects in the virtual space can be set at far, intermediate, and near distances, as well as the left and right frontal, diagonal, and side directions, depending on the task [11]. Trunk training was performed using the VR device customized to enable anterior pelvic tilt, trunk extension, and lower limb loading on the reaching side.

Outcome and follow-up

Table 1 presents the changes observed in each period in the order of B1, A, and B2 (B1-Baseline/A-B1/B2-A). The 10 MWT and number of steps at the end of periods B1, A, and B2 were -5.9 s (-9 steps), 0.1 s (1 step), and -2.5 s (-3 steps), respectively. The hip extension range of motion (right/left) during gait at the end of periods B1, A, and B2 was 7.1°/9.4°, 2.0°/-3.5°, and-1.0°/18.0°, respectively. The step length (right/left) was 11.3 cm/2.7 cm, -4.8 cm/0 cm, and 8.4 cm/4.2 cm, respectively, indicating an increase in step length and a decrease in gait time. The seated pelvic tilt at the end of periods B1, A, and B2 was 11.1°, 1.1°, and 5.5°, respectively. The standing pelvic shift at the end of periods B1, A, and B2 was -5.3 cm, -0.4 cm, and -1.9 cm, respectively.

**Table 1:** Time-series data for the outcomes Abd: abduction; BBS: Berg Balance Scale; DF: dorsiflexion; EO: external oblique muscle; ext: extension; flex: flexion; FRT: functional reach test; MF: multifidus muscle; PM: psoas major muscle; TrA: transverse abdominal muscle; IO: internal oblique muscle; MWT: 10-meters walking test

Variables		Baseline	B1		A		B2	
			Value	B1 － Baseline	Value	A－B1	Value	B2－A
Gait parameters	10 MWT (sec)	20.5	14.6	-5.9	14.7	0.1	12.2	-2.5
	Step (number)	38	29	-9	30	1	27	-3
	Rt hip ext angle (°)	-15.9	-8.8	7.1	-6.8	2	-7.8	-1
	Lt hip ext angle (°)	-15.5	-6.1	9.4	-9.6	-3.5	-7.8	1.8
	Rt trunk tilt angle (°)	12.9	13.8	0.9	10.5	-3.3	12.9	2.4
	Lt trunk tilt angle (°)	14.3	9.1	-5.2	7.2	-1.9	8.7	1.5
	Rt step length (㎝)	17.7	29	11.3	24.2	-4.8	32.6	8.4
	Lt step length (㎝)	31.5	34.2	2.7	34.2	0	38.4	4.2
Posture	Sitting pelvic tilt (°)	-15.4	-4.3	11.1	-3.2	1.1	2.3	5.5
	Standing pelvic shift	7.6	2.3	-5.3	1.9	-0.4	0	-1.9
Trunk functions	Rt MF (㎠)	3.8	3.8	0	4.2	0.4	4.4	0.2
	Lt MF (㎠)	5.7	5.8	0.1	5.5	-0.3	6	0.5
	Rt PM (㎠)	5.3	5.4	0.1	5.4	0	5.6	0.2
	Lt PM (㎠)	5.3	6.4	1.1	5.1	-1.3	6.2	1.1
	Rt EO (㎜)	2	1.7	-0.3	1.2	-0.5	1.7	0.5
	Lt EO (㎜)	1.5	1.3	-0.2	1.4	0.1	1.3	-0.1
	Rt IO (㎜)	1.9	2.4	0.5	2.1	-0.3	2	-0.1
	Lt IO (㎜)	3	2.3	-0.7	2.2	-0.1	2.9	0.7
	Rt TrA (㎜)	1.5	1	-0.5	1.1	0.1	1.8	0.7
	Lt TrA (㎜)	1.9	1.4	-0.5	1.8	0.4	2.4	0.6
	Maximum pelvic anterior tilt (°)	-8	0	8	0	0	3	3
Performance	Five Times Sit to Stand Test (sec)	17.4	10.6	-6.8	9.9	-0.7	9.1	-0.8
	FRT (㎝)	7.5	13.8	6.3	14.1	0.3	19.5	5.4
	BBS (points)	29	35	6	35	0	39	4
L/E functions	Hip flex range (°)	100	100	0	100	0	100	0
	Hip ext range (°)	25	25	0	25	0	25	0
	Knee ext range (°)	0	0	0	0	0	0	0
	Ankle DF range (°)	15	15	0	15	0	15	0
	Hip ext strength (KgF)	3.4	3.4	0	3.5	0.1	3.6	0.1
	Hip Abd strength (KgF)	3.8	3.8	0	4	0.2	4.2	0.2
	Knee ext strength (KgF)	3.8	3.8	0	4.1	0.3	5.1	1
	Rt maximum weight shift (Kg)	33	33	0	33	0	33	0
	Lt maximum weight shift (Kg)	33	33	0	32	-1	33	1

**Figure 4:**
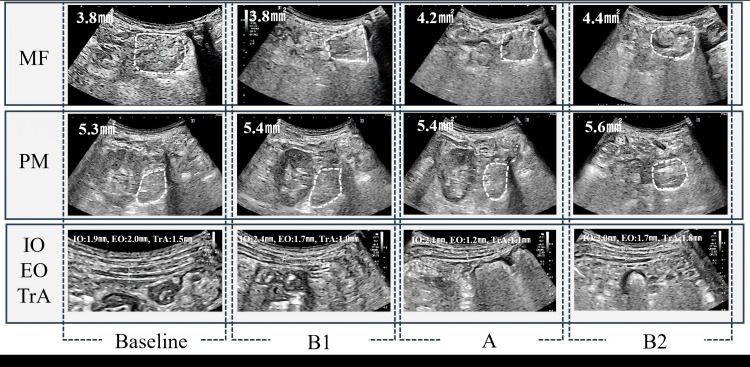
Changes in cross-sectional area and thickness of the trunk muscles EO: external oblique abdominal muscle; IO: internal oblique abdominal muscle; MF: multifidus muscle; PM: psoas major muscle; TrA: transverse abdominal muscle

These findings indicate an improvement in the posterior pelvic tilt alignment and pelvic forward displacement. The maximum anterior pelvic tilt angle in the trunk function index improved to 8.0°, 0°, and 3.0° at the end of periods B1, A, and B2, respectively. The Five Times Sit to Stand Test conducted to assess the performance was shortened to -6.8 seconds, -0.7 seconds, and -0.8 seconds at the end of periods B1, A, and B2, respectively. The patient required assistance from a nurse to ambulate using a walker during the initial period of hospitalization; however, she was able to ambulate independently at the end of the B2 period and regained the pre-injury gait modality. Notably, no changes in the cross-sectional area of the trunk muscle, muscle thickness (Figure 4), or lower extremity function were observed over the entire period.

## Discussion

Our patient was able to walk independently two months after sustaining a femoral neck fracture following the implementation of a VR-guided trunk training intervention. The maximum anterior pelvic tilt angle and sitting and standing postures improved; the hip extension range of motion and step length during gait increased; and the 10 MWT decreased during the VR-guided intervention period. These findings indicate the importance of implementing interventions targeting the trunk muscles and the effectiveness of VR-guided trunk training in patients with femoral neck fractures.

The use of mediVR KAGURA improves gait speed without inducing any changes in lower limb muscle strength in patients with disuse syndrome [9] and reduces plantar sensation, as well as trunk sway, in patients with hallux valgus [10]. However, the effect of mediVR KAGURA among older adults with impaired trunk function after femoral neck fracture remains unclear. The present report revealed that trunk movement, posture, balance, and gait can be improved without trunk muscle hypertrophy, even in patients with femoral neck fractures and impaired trunk function.

Lumbar kyphosis has been associated with reduced trunk extensor strength [12] and dynamic balance [13]. The multifidus and psoas major muscles are involved in lumbar lordosis [14]. Our patient showed improvement in sitting alignment and maximum anterior pelvic tilt angle. Moreover, the time for the Five Times Sit to Stand Test of repetitive pelvic anterior-posterior tilt movement also exhibited a substantial reduction. These findings suggest an improvement in the function of the multifidus and psoas major muscles. Flexed trunk gait observed before implementing the intervention was thought to increase hip extensor loading [15] and decrease the gait speed. The improvement in trunk muscle function was accompanied by a decrease in the forward tilt of the trunk during walking, an increase in hip extension, and a decrease in the number of steps taken. Consequently, the gait time was shortened, with no significant change in lower extremity function.

The improvement in dynamic balance ability affects gait [16], and patients can attain independent gait with a walker. Normal reach exercises are performed by obtaining visual feedback of the target point and body position information [17]. However, the reaching motion performed using mediVR KAGURA provides auditory, tactile, and visual sensory feedback without visual recognition of the body [9,11,18]. The dual-task also showed improvements in cognitive function [19]. These results could be used to formulate interventions for predictive postural control. In addition, the appropriate adjustment of the task (distance, speed, and frequency) and the pleasant, game-like approach made the exercise more feasible than normal reaching [11].

Limitations

This case report presented the findings of a single case. Further randomized controlled trials must be conducted to evaluate the effectiveness of VR-guided trunk training. Furthermore, although the improvement in trunk function was related to postural control, no change in the cross-sectional area or thickness of the trunk muscles was observed. Thus, this hypothesis could not be verified. The intervention period should be extended to account for muscle hypertrophy.

## Conclusions

Decreased trunk function has been identified as a factor contributing to decreased gait ability after femoral neck fracture. Our findings indicate that VR-guided seated trunk training may be an effective intervention for improving trunk function and gait ability. Seated trunk training using VR is a safe and comfortable intervention that can be implemented in clinical practice to facilitate effective trunk training in older adults. Seated reach training using VR equipment improved trunk flexion during gait and reduced walking time. VR-guided trunk training can effectively improve trunk muscle function and gait performance after femoral neck fractures.
